# Equivalent modeling of PMSG-based wind power plants considering LVRT capabilities: electromechanical transients in power systems

**DOI:** 10.1186/s40064-016-3700-5

**Published:** 2016-11-29

**Authors:** Ming Ding, Qianlong Zhu

**Affiliations:** Anhui New Energy Utilization and Energy Saving Laboratory, Hefei University of Technology, No. 193 Tunxi Road, Baohe District, Hefei, 230009 Anhui Province China

**Keywords:** Equivalent WPP model, PMSG, LVRT capabilities, DC link voltage, Chopper circuit, Coordinated power control

## Abstract

Hardware protection and control action are two kinds of low voltage ride-through technical proposals widely used in a permanent magnet synchronous generator (PMSG). This paper proposes an innovative clustering concept for the equivalent modeling of a PMSG-based wind power plant (WPP), in which the impacts of both the chopper protection and the coordinated control of active and reactive powers are taken into account. First, the post-fault DC link voltage is selected as a concentrated expression of unit parameters, incoming wind and electrical distance to a fault point to reflect the transient characteristics of PMSGs. Next, we provide an effective method for calculating the post-fault DC link voltage based on the pre-fault wind energy and the terminal voltage dip. Third, PMSGs are divided into groups by analyzing the calculated DC link voltages without any clustering algorithm. Finally, PMSGs of the same group are equivalent as one rescaled PMSG to realize the transient equivalent modeling of the PMSG-based WPP. Using the DIgSILENT PowerFactory simulation platform, the efficiency and accuracy of the proposed equivalent model are tested against the traditional equivalent WPP and the detailed WPP. The simulation results show the proposed equivalent model can be used to analyze the offline electromechanical transients in power systems.

## Background

To investigate the effects of ongoing changes in power systems with the increasing penetration of wind power, an accurate and reasonable WPP model is essential. A detailed WPP model, in which the dynamics of each wind turbine and the internal network are both fully represented, is not suitable as it could significantly increase the order of the power system’s mathematical model to be solved and results in a much longer simulation time. As a result, the equivalent WPP model is generally recommended for reflecting the collective response of the entire WPP in large power systems.

Equivalent WPP models using aggregated wind turbines are classified as either a single-machine representation or a multiple-machine representation (Muljadi and Ellis 2010). In the first case, the actual WPP is modeled as a unique re-scale wind turbine. This is rational when all wind turbines are operating under the identical condition; however, that might not be the case in real-world system operations. Nowadays, in order to improve the conformity of the single-machine representation with the actual WPP, numerical identification methods are applied to optimize the parameters of equivalent models. The optimized objects include generator (Elizondo et al. 2011; Erlich et al. 2012), power converter (Jin et al. 2014) and passive frequency-dependent network (Hussein et al. 2013).

In the second case, a study of the multiple-machine representation focuses on selecting reasonable clustering indices by quantifying and abstracting key features of wind turbines. The coherency method is presented by clustering wind turbines according to different wind speeds (Fernández et al. 2009; Zubia et al. 2012). In the research work (Ali et al. 2013) the wind direction is taken into consideration. Then, generator speed, stator voltage, *q*-axis component of stator current, and real-time active power are also implemented as clustering indices (Zou et al. 2015). To represent the power loss on the collective network within the WPP, Muljadi et al. (2006) introduce a method of calculating the equivalent parameters of line and cable. Moreover, Cheng et al. (2011) analyze the impact of line impedance on diversity in the voltage profile of each wind turbine, and describe a voltage-profile-based approach to develop a multiple-machine aggregated WPP. In addition, a clustering index with consideration to the influence of fault types on coherency is depicted (Zhu et al. 2014). However, most of the aforementioned studies placed emphasis on the doubly fed induction generator (DFIG)-based WPP.

With the rapid development of wind power generation and power electronics, the PMSG has become the current trend based on specific characteristics, such as its gearless drive train, high LVRT capability, and its ability to decouple the generator from the grid fault. At present, the study of multiple-machine representation for PMSG-based WPPs only takes into account diversity in wind speeds (Abbes et al. 2015; Conroy and Watson 2009). Furthermore, since the speed changes in both the wind turbine and generator are negligible during the voltage dip, the simplified equivalent PMSG-based WPP models are recommended, in which the drive train, the generator and the machine side converter (MSC) are omitted (Conroy and Watson 2009; Neumann et al. 2015). Although the chopper circuit is taken into account, these simplified models with single-machine representation or multiple-machine representation cannot accurately reflect the diversity in chopper circuit conductions within the WPP. As for DFIG-based WPPs, the traditional equivalent WPP model built based on wind speeds experiences large discrepancies due to its failure to represent the diversity in crowbar circuit conductions (Brochu et al. 2011). Similarly, the PMSGs are equipped with the chopper circuits, and the diversity in chopper circuit conductions should also be abstracted while clustering the PMSGs.

The goal of this paper is to highlight an equivalent modeling of PMSG-based WPPs considering LVRT capabilities. Taking into account the coordinated active and reactive power control strategies, the post-fault DC link voltage is calculated based on the pre-fault wind energy and the terminal voltage dip. The chopper circuit is triggered once the calculated voltage reaches the chopper circuit threshold. When the calculated voltage is between the chopper circuit threshold and the normal value, it means the DC link voltage continuously rises without triggering the chopper circuit. For the rest scenario, it signifies that the DC link voltage fluctuates around the normal value. Eventually, the PMSGs will be divided into three groups according to the range in which DC link voltage changes, and thus to establish the equivalent model of PMSG-based WPPs. Using the PowerFactory DIgSILENT software platform, we verified the efficiency and accuracy of the proposed equivalent WPP.

## Background on LVRT scheme of a PMSG

**Fig. 1:**
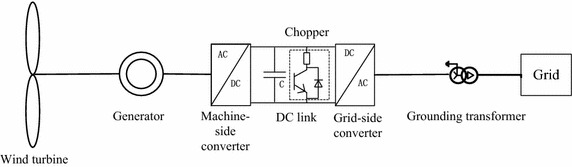
Main electrical and mechanical components of PMSG

A PMSG is connected to the grid through a full scale power converter, with which the LVRT capability can be achieved. The primary electrical and mechanical components are shown in Fig. 1.

Enhancement of the LVRT capability for PMSGs can be achieved through two approaches: hardware protection and control action. As for the unbalanced active power delivered by the generator to the DC link, the method for improving control strategies on the MSC, wherein the rotor of the wind turbine is used to store the energy, is feasible, but the response rate of the method is relatively slow owing to the high inertia of the wind turbine. Therefore, the DC link chopper circuit is normally equipped to absorb any excess active power. Meanwhile, in order to offer the network support, the control pattern of the grid side converter (GSC) is usually modified to preferentially support reactive power during this fault.

### Chopper circuit

During the electromechanical transient process, the wind energy of the wind turbine is approximately constant, while the output active power of the GSC reduces under a voltage dip. As a result, the DC link voltage will continuously rise due to this unbalanced active power. The dynamic equation of the DC link voltage can be described as:

1 $$\Delta P_{dc} = P_{t} - P_{s} = CU_{dc} \frac{{dU_{dc} }}{dt}$$

where $P_{s}$ is the output active power of the GSC, $C$ is the capacitance of the DC link, $U_{dc}$ is the DC link voltage.

In order to prevent the DC link voltage from rising too roughly, the brake chopper is a simple protection device that shorts the DC link through a power resistor when the DC link voltage exceeds a fixed threshold. As shown schematically in Fig. 1, an insulated gate bipolar rectifier (IGBT) chopper circuit is used to rapidly engage and disengage the resistor. The chopper works on a hysteresis band, i.e., the turn-off voltage (1.05 pu) is set below the turn-on threshold (1.06 pu).

### Coordinated power control of the GSC

**Fig. 2:**
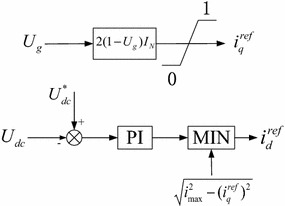
Coordinated power control of the GSC

Under the normal voltage condition, the active power priority pattern is usually adopted to maximize the utility of natural wind energy. When a fault occurs, however, the control pattern of the GSC should be transferred to the coordinated power control strategy in which the output reactive power is prior to the output active power and the reference *d*-current component $i_{d}^{ref}$ of the GSC is set as the minimum value between the current commend of the PI controller for DC link voltage regulation and $\sqrt {i_{\rm{max} }^{2} - (i_{q}^{ref} )^{2} }$, shown in Fig. 2. So it can fully enhance the LVRT capability of PMSGs and support the grid voltage.

We have noted that the grid voltage cannot regain the normal value before a fault clearance, though the reactive powers are provided by PMSGs. Accordingly, the reference *q*-current component $i_{q}^{ref}$ of the GSC could be adjusted based on the voltage dip to support the appropriate reactive power, instead of the current command of the PI controller for reactive power regulation. In this paper, the reference *q*-current component $i_{q}^{ref}$ of the GSC is calculated based on the literature (State Grid Corporation of China 2009):

2 $$i_{q}^{ref} = 2(1 - U_{g} )I_{N} \quad (0.2\,{\text{pu}} \le U_{g} \le 0.9\,{\text{pu}})$$

where, $U_{g}$ is the terminal voltage dip (pu), $I_{N}$ is the rated current of the GSC (pu).

## Multiple-machine representation method for PMSG-based WPPs

### Clustering principle

When the grid voltage dips, the brake chopper results in negligible speed changes in both the wind turbine and generator, so the speed index cannot completely represent the transient characteristics of the PMSGs. However, as the GSC switches to a reactive power priority control pattern, the DC link voltage undergoes a significant change, and the changes between different operating conditions of PMSGs are obviously different. For the PMSG subjected to a high wind speed, the DC link voltage may continue to rise until the threshold of the brake chopper, and then the chopper circuit is triggered. As for the PMSG subjected to a low wind speed, the DC link voltage may only show a slight fluctuation in the neighborhood of the normal value (1.0 pu), since the unbalanced active power delivered by the generator to the DC link is relatively small and the DC link voltage can be effectively adjusted by the GSC.

It should be noted that different DC link voltage changes can lead to different conduction statuses of the chopper circuits. The characteristics of the chopper circuit conduction can reflect both the power loss consumed by the chopper resistor and the status of the DC capacitor energy storage. In addition, when DC link voltage rises or fluctuates within the fault duration, it represents the adjustment ability of the GSC and the active-power behavior of the PMSG. A continued rise in the DC link voltage indicates the GSC has already lost its active power control ability due to its current limit. In this case, the PMSG will maintain the maximum output active power within the fault duration, and has an active power pulse after the fault clearance. The DC link voltage fluctuating near the normal value reveals that the GSC still has its active power control ability, so the output active power will only fluctuate near its pre-fault value within the fault duration and will smoothly recover to a new steady state after the fault clearance.

Therefore, the different changes in DC link voltages are selected as a clustering principle, e.g., PMSGs are divided into three groups at most: Group 1) chopper circuit conduction within the fault duration; Group 2) the DC link voltage continuously rising without reaching the chopper circuit threshold within the fault duration; Group 3) the DC link voltage fluctuating in the neighborhood of the normal value within the fault duration.

The quantization of the DC link voltage fluctuation in the neighborhood of the normal value will be described in “Clustering index calculation” section.

### Clustering index calculation

In general, the wind energy of the wind turbine is considered constant in the electromechanical transient analysis, and the terminal voltage dip of the PMSG is also approximately constant after a rise owed to the reactive power supported by PMSGs. Therefore, the post-fault DC link voltage can be directly calculated based on the pre-fault wind energy and the terminal voltage dip, as follows:

3 $$\frac{1}{2}CU_{dc}^{2} (t_{1} ) - \frac{1}{2}CU_{dc}^{2} (t_{0} ) = \left( {P_{t} - U_{g} i_{d}^{ref} S_{B} } \right)\left( {t_{1} - t_{0} } \right)$$

where $t_{0}$, $t_{1}$ are the fault initial time and fault clearing time, respectively. $S_{B}$ is the base value of the GSC.

It is worth noting that the reference *d*-current component $i_{d}^{ref}$ of the GSC should choose the minimum value between the current command of the PI controller for DC link voltage regulation and $\sqrt {i_{\rm{max} }^{2} - (i_{q}^{ref} )^{2} }$, as shown in Fig. 2. Considering the engineering operability in the electrical data collection process, however, the value of $i_{d}^{ref}$ is always set as $\sqrt {i_{\rm{max} }^{2} - (i_{q}^{ref} )^{2} }$ in the Eq. (3). As a result, if the right side of the Eq. (3) is not lower than 0, the GSC still fails to meet the adjusted requirement of the PI controller for DC link voltage regulation, even though the value of the reference *d*-current component $i_{d}^{ref}$ has been set as its current limit. Therefore the DC link voltage will rise. If the right side of the Eq. (3) is lower than 0, the DC link voltage will fluctuate near the normal value since the GSC can meet the adjusted requirement of the PI controller for DC link voltage regulation. In this paper, when the DC link voltage rises or fluctuates, it can be distinguished based on the relationship between the post-fault DC link voltage and the normal value, e.g., if the post-fault DC link voltage calculated based on Eq. (3) is lower than the normal value, it means the DC link voltage will fluctuate near the normal value, otherwise it will rise.

### Parameters calculation of equivalent WPP model

The method of calculating the equivalent parameters of wind, generator, grounding transformer and collection line can be referred to in the literature (Zou et al. 2015). Here, the equivalent parameters of the DC link capacitance and chopper resistor are calculated as follows:

4 $$\left\{ {\begin{array}{*{20}l} {C_{eq\_i} = m_{i} C} \hfill \\ {r_{eq\_i} = r_{ch} /m_{i} } \hfill \\ \end{array} } \right.$$

where *r*_*ch*_ is the chopper resistor, *m*_*i*_ is the number of PMSGs within the group *i*. The subscript* eq* denotes the equivalent parameter.

## Results and discussion

The proposed equivalent WPP was tested by comparing its transient response against the traditional equivalent WPP and the detailed WPP.

### Model development

**Fig. 3:**
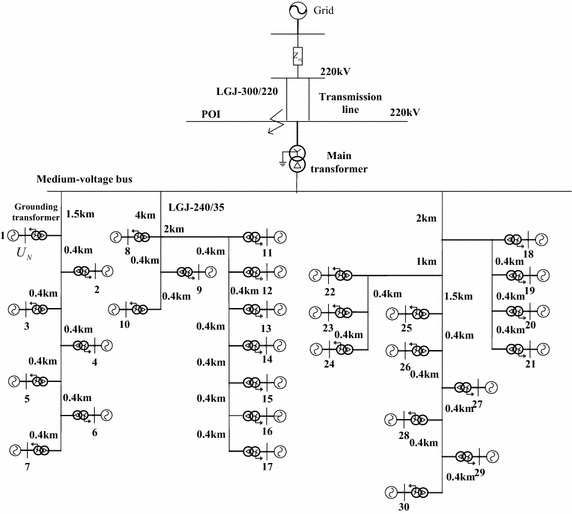
Structure of PMSG-based WPP

A detailed WPP consisting of $30 \times 2$ MW PMSGs (PMSG_1—PMSG_30) was set up in the simulation platform DIgSILENT/Power Factory, as shown in Fig. 3. Each PMSG is connected to the collector system through a 0.69/35 kV grounding transformer, and the output is delivered to the 35/220 kV main transformer via 50 km transmission lines. The interval between two PMSGs is shown in Fig. 3, and model parameters are given in Table 4 in “Appendix”. Prior to the faults, PMSGs operated with a unity power factor.

Compared with the simulation results of the detailed WPP, error indices are defined as:

5 $$E_{a} = \frac{1}{n}\sum\limits_{i = 1}^{n} {\left| {Y_{fi} (k) - Y_{i} (k)} \right|}$$

6 $$E_{r} = \frac{1}{n}\sum\limits_{i = 1}^{n} {\left| {\frac{{Y_{fi} (k) - Y_{i} (k)}}{{Y_{i} (k)}}} \right|}$$

where $Y_{i} (k)$ and $Y_{fi} (k)$ are the output variables of the complete collector system for the equivalent WPPs and the detailed WPP, respectively, and n stands for the number of PMSGs within the WPP.

In this paper, the proposed equivalent WPP model is named as the equivalent model A, and the traditional equivalent WPP model, based on the principle that PMSGs are divided into groups according to wind speed, is named as equivalent model B.

### Comparison between calculated and simulated DC link voltages

In order to test the accuracy of the calculation method mentioned in “Clustering index calculation” section, the post-fault DC link voltages calculated based on the Eq. (3) are compared with the simulated ones.

In the rated power range, the active powers of 30 PMSGs were randomly generated. A three-phase short-circuit fault was applied at the POI (point of interconnection), as shown in Fig. 3. The 150-ms fault starts at 0.1 s, and then the POI voltage dips to 0.6 pu. In the case, the triggering signal of the chopper circuit is disabled. The calculated and simulated DC link voltages at the fault clearing time are listed in Table 1.

**Table 1:** DC link voltages and group no.

PMSG	P (MW)	Calculated result (pu)	Simulated result (pu)	Group no. based on calculated result	Group no. based on simulated result	PMSG	P (MW)	Calculated result (pu)	Simulated result (pu)	Group no. based on calculated result	Group no. based on simulated result
1	0.25	0.9476	0.9944	3	3	16	1.27	1.0837	1.0842	1	1
2	0.58	1.0003	1.0076	2	2	17	0.86	1.0158	1.0222	2	2
3	0.35	0.9539	0.9944	3	3	18	0.52	0.9617	0.9902	3	3
4	1.06	1.0710	1.0713	1	1	19	0.56	0.9662	0.9882	3	3
5	1.47	1.1322	1.1306	1	1	20	0.68	0.9857	0.9980	3	3
6	1.88	1.1911	1.1887	1	1	21	0.94	1.0293	1.0342	2	2
7	1.83	1.1831	1.1807	1	1	22	0.82	0.9962	1.0039	3	2
8	1.65	1.1877	1.1844	1	1	23	0.72	0.9771	0.9919	3	3
9	1.03	1.0923	1.0910	1	1	24	1.00	1.0245	1.0304	2	2
10	1.93	1.2245	1.2209	1	1	25	1.25	1.0519	1.0552	2	2
11	0.43	0.9593	0.9925	3	3	26	0.54	0.9244	0.9958	3	3
12	0.32	0.9335	0.9948	3	3	27	0.80	0.9688	0.9887	3	3
13	0.14	0.8948	0.9954	3	3	28	0.98	1.0009	1.0086	2	2
14	0.72	1.0012	1.0070	2	2	29	1.01	1.0048	1.0117	2	2
15	0.91	1.0267	1.0316	2	2	30	1.56	1.0914	1.0924	1	1

As can be seen from Table 1, for the PMSGs whose simulated DC link voltages are higher than the normal value, the calculated DC link voltages are very close to the simulated ones. This effectively divides most of the above PMSGs into Group 1 or Group 2 based on the proposed calculation method, instead of the detailed WPP’s simulation results, even though there is one PMSG, i.e., PMSG_22, which has been wrongly divided into Group 3. As for the PMSGs whose simulated DC link voltages are lower than the normal value, the calculated results are lower than the simulated ones. This is because the value of $i_{d}^{ref}$ is always set as $\sqrt {i_{\rm{max} }^{2} - (i_{q}^{ref} )^{2} }$, instead of the minimum value between the current command of the PI controller for DC link voltage regulation and $\sqrt {i_{\rm{max} }^{2} - (i_{q}^{ref} )^{2} }$. However, these errors will not make the mistake of dividing the above PMSGs into the wrong group because both the calculated and simulated DC link voltages are lower than the normal value. Based on the grouping principle, the PMSGs should be divided into Group 3 if the DC link voltage at the fault clearing time is lower than the normal value.

**Fig. 4:**
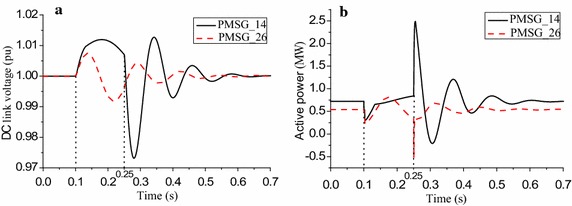
**a** DC link voltages, **b** active powers of PMSGs

It is noteworthy that the slight ascent and slight fluctuation of DC link voltages are two different transient processes, as shown in Fig. 4a. The slight ascent means the DC link voltage is always higher than the normal value from fault initiation to clearance, while the slight fluctuation means the DC link voltage fluctuates both higher and lower than the normal value during the above period. The two different DC link voltage transient processes reflecting on the output of PMSGs mean not only the fluctuations of the active powers are different, but the pulse directions of the active powers after fault clearing are different as well, as shown in Fig. 4b. Different active power trends further verify the reasonableness of the proposed clustering principle.

### Different POI voltage dips

The active powers of 30 PMSGs are listed in Table 1. A three-phase short-circuit fault was applied at the POI, as shown in Fig. 3. The 150-ms fault starts at 0.1 s. In order to test the suitability of the proposed equivalent method for different voltage dips, we changed the short circuit grounding impedance to successively adjust the POI voltage dip within the range of 0.2–0.9 pu with the step of 0.1 pu.

**Fig. 5:**
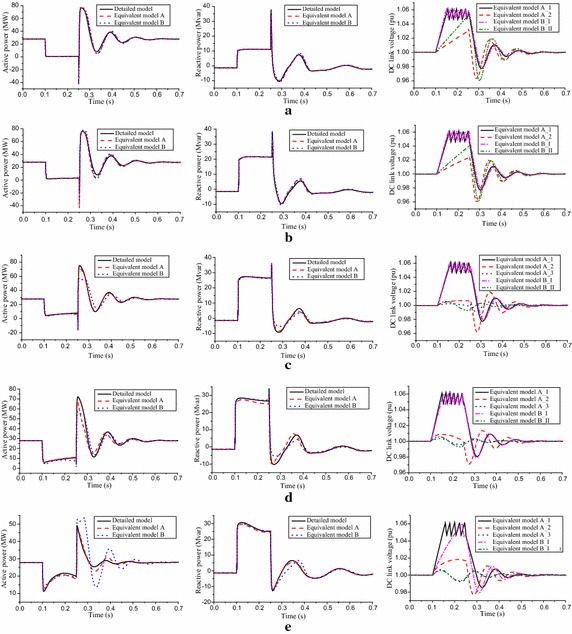
Dynamic response of the WPPs. **a** $U_{POI} = 0.2\,{\text{pu}}$, **b** $U_{POI} = 0.3\,{\text{pu}}$, **c** $U_{POI} = 0.4\,{\text{pu}}$, **d** $U_{POI} = 0.5\,{\text{pu}}$, **e** $U_{POI} = 0.6\,{\text{pu}}$

**Fig. 6:**
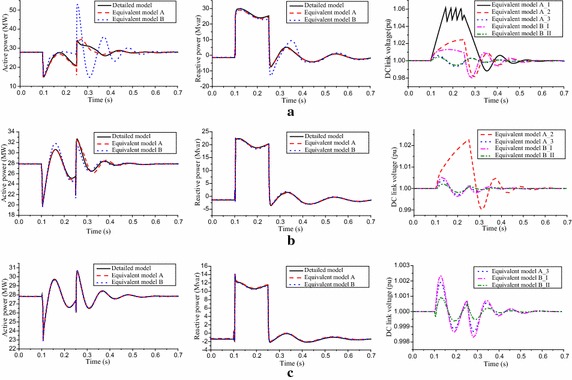
Dynamic response of the WPPs. **a** $U_{POI} = 0.7\,{\text{pu}}$, **b** $U_{POI} = 0.8\,{\text{pu}}$ and **c** $U_{POI} = 0.9\,{\text{pu}}$

Figures 5 and 6 illustrates the behavior of the active and reactive powers of the POI for the two equivalent models and the detailed WPP following the application of the three-phase short-circuit faults when the POI voltage ranges from 0.2 to 0.9 pu. The DC link voltages of equivalent PMSGs within the two equivalent models are also shown in Figs. 5 and 6. As can be seen from Fig. 5a, b, the collective response of the two equivalent models and the detailed WPP match, and the errors of curve fitting lie in horizontal displacement and amplitude. This is because the POI voltage dips are very severe, so the transient characteristics of PMSGs are dominated by the fault factor, instead of the active power and electrical distance. As a result, transient response characteristics of each PMSG within the detailed WPP are similar, and the transient response characteristics of each equivalent PMSGs within the two equivalent models are also similar. With the fault mitigation, the advantage of the equivalent model A is fully performed, as seen in Figs. 5c–e and 6a−c.

Combined with the group results in Table 2 and the DC link voltage response of the equivalent models in Figs. 5 and 6, we will explain why the traditional equivalent model has large power discrepancies when it is based on the principle that PMSGs are divided into groups according to wind speed. As for the Fig. 5e, depending upon the group results of the equivalent model A, there are 9 PMSGs with chopper circuit conductions, while the simulation results of the equivalent model B mean there are 21 PMSGs conducting the chopper circuits. Under the same input wind energy, more PMSGs with chopper circuit conductions mean greater energy loss in chopper resistors and greater energy storage in DC capacitors. Accordingly, the active power pulse of the equivalent model B is enhanced because more stored energy in DC capacitors has been released after the fault clearance. As for the Fig. 6b, the number of PMSGs with DC link voltage fluctuation is 28, depending on the group results of the equivalent model A, while the simulation results of the equivalent model B mean that all of 30 PMSGs’ DC link voltages fluctuate near the normal value. More PMSGs with DC link voltage fluctuations mean the output active power fluctuations are enhanced within the fault duration, while the amplitude of the active power pulse is reduced after the fault clearance.

**Table 2:** Group results and model error indices

$U_{POI}$ (pu)	Group results					$\Delta P$ (%)		$\Delta Q$ (Mvar)	
	Equivalent A			Equivalent B		Equivalent A	Equivalent B	Equivalent A	Equivalent B
	Group 1	Group 2	Group 3	Group I	Group II				
0.2	26	4	×	21	9	6.41	7.64	0.329	0.345
0.3	25	5	×	21	9	6.95	7.11	0.456	0.394
0.4	23	6	1	21	9	3.39	12.11	0.481	0.824
0.5	16	12	2	21	9	8.34	10.41	1.208	0.907
0.6	9	9	12	21	9	3.44	12.49	0.475	1.039
0.7	4	6	20	21	9	2.23	14.27	0.367	1.425
0.8	×	2	28	21	9	0.69	1.13	0.112	0.199
0.9	×	×	30	21	9	0.041	0.036	0.129	0.068

× Stands for there are no PMSGs in the group

During the fault period, PMSGs are switched to a reactive priority control strategy in which the output reactive power depends on the terminal voltage dip, which relates to the electrical distance. Due to the reasonable group result, the equivalent model A is also more accurate in the reactive power under the same collection system equivalent method.

Table 2 also lists the equivalent accuracy of the equivalent WPPs compared to the detailed WPP regarding error indices. The proposed equivalent method divides the PMSGs by the diversity in the DC link voltage changes, and the coordinated power control strategy and the conduction status of the chopper circuit are also taken in account, thus significantly improving the accuracy of the equivalent model.

**Fig. 7:**
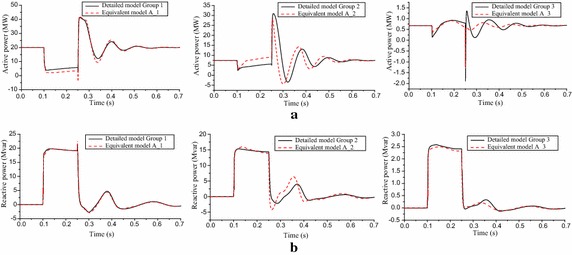
Dynamic response of each group when the POI voltage dips to 0.5 pu. **a** Output active power. **b** Output reactive power

It should be mentioned that the proposed model has significant active and reactive power discrepancies when the POI voltage dips to 0.5 pu. Comparing the active and reactive powers of each group before and after the equivalence, as shown in Fig. 7, we can see that the equivalent PMSG representing the Group 2 mainly reduces the conformity of the proposed model with the detailed WPP. This is due to the imprecision in parameters of equivalent collector system. In fact, the method for collector system equivalence relies on the assumption that the current injection from all PMSGs is identical in angle, and adopts approximately the POI voltage to calculate the output current of each PMSG, instead of the actual terminal voltage (Muljadi and Ellis 2010; Zou et al. 2015; Zhu et al. 2014). When the POI voltage dips to 0.5 pu, the parameter error caused by the two simplifications is enlarged. Therefore, the proposed method has a different response. In order to improve the conformity of the proposed model with the detailed WPP in this case, further research might be needed to optimize the parameters of equivalent collector system.

To further verify the effectiveness of the proposed equivalent method, the following cases were carried out.

### Different output active powers and transfer coefficients of wind energy

There are three feeders in the detailed WPP, as shown in Fig. 3, and they are successively numbered from left to right. For example, the transfer coefficient of wind energy is equal to 0.9 pu, and then the output active powers of the PMSGs in Feeder 1, Feeder 2 and Feeder 3 were randomly generated within the range of 1–0.9, 0.9–0.81 and 0.81–0.729 pu, respectively. We provided three different transfer coefficients of wind energy, i.e., 0.7, 0.8, and 0.9 pu, to modify the difference in active powers of the PMSGs within the WPP.

Limited to the length of the article, we have omitted the transient behavior of the active and reactive powers of the POI for the two equivalent WPPs and the detailed WPP, and only list the model errors in Table 3.

**Table 3:** No. of groups and model error indices

Transfer coefficient of wind energy	$P_{wind}$ (MW)	No. of groups		$\Delta P$ (%)		$\Delta Q$ (Mvar)	
		Equivalent A	Equivalent B	Equivalent A	Equivalent B	Equivalent A	Equivalent B
0.7	0.4	1	2	1.45	0.17	0.120	0.101
	0.6	1	2	4.53	3.97	0.183	0.098
	0.8	2	2	3.38	5.67	0.126	0.212
	1.0	2	2	2.35	6.44	0.107	0.239
	1.2	3	2	1.17	2.51	0.088	0.131
	1.4	3	2	1.93	5.17	0.179	0.374
	1.6	3	2	1.83	10.88	0.193	0.999
	1.8	3	2	2.45	7.20	0.251	0.763
	2.0	3	2	2.68	3.13	0.309	0.515
0.8	0.4	1	2	0.42	0.18	0.056	0.009
	0.6	1	2	4.85	3.52	0.214	0.135
	0.8	2	2	1.94	6.45	0.081	0.216
	1.0	2	2	2.14	3.78	0.126	0.290
	1.2	3	2	2.74	9.37	0.218	0.790
	1.4	3	2	2.00	7.97	0.201	0.696
	1.6	3	2	1.92	3.34	0.313	0.392
	1.8	2	2	2.98	4.58	0.370	0.526
	2.0	2	2	2.26	3.41	0.268	0.363
0.9	0.4	1	2	0.19	0.17	0.025	0.013
	0.6	2	2	3.65	8.13	0.186	0.326
	0.8	2	2	4.94	4.94	0.257	0.257
	1.0	2	2	1.93	8.40	0.171	0.671
	1.2	3	2	0.68	2.08	0.103	0.237
	1.4	2	2	0.78	3.18	0.126	0.264
	1.6	2	2	1.42	1.87	0.207	0.267
	1.8	2	2	1.06	2.39	0.204	0.350
	2.0	1	2	2.08	1.39	0.394	0.287

With the proposed equivalent method, conformity with the detailed WPP of the active and reactive powers of the equivalent WPP is generally improved except a few cases. These cases are related to the following situations: (1) the group number of the equivalent model A is lower than that of the equivalent model B. This phenomenon occurs in the event that all of 30 PMSGs’ DC link voltages reach the chopper circuit threshold or fluctuate near the normal value. Based on the proposed equivalent method, these 30 PMSGs are clustered into one group. However, the 30 PMSGs are clustered into two groups using the traditional equivalent method in which the clustering principle is dependent on the diversity in wind speeds. Simulation results reveal that both the equivalent model A and B can reflect the phenomenon that all of 30 PMSGs’ DC link voltages reach the chopper circuit threshold or fluctuate near the normal value, but equivalent model B also takes into account the diversity in wind speeds. On the basis of increasing the order of equivalent model, the equivalent model B improves its precision somewhat. (2) The equivalent model A and B have a same clustering result, as can be seen when wind energy and transfer coefficient are 0.8 MW, 0.9 pu, respectively.

It should be noted that the model error indices are obviously reduced when the equivalent model A performs better than the equivalent model B. However the difference in the active power errors between the two equivalent models are all about 1% when the equivalent model A performs worse than the equivalent model B. Based on a credibility analysis of the power systems’ dynamic simulation, the swing amplitude is generally utilized to evaluate whether a system can achieve new stability after the fault clearance. Accordingly, we selected the active power pulse error at POI after the fault clearance as an indicator to evaluate the credibility of the proposed equivalent model, as follows:

7 $$E_{M} = \frac{{M_{f} - M}}{M}$$

where $M_{f}$ and $M$ are the amplitudes of the active power pulses after the fault clearance for the equivalent model and the detailed WPP, respectively.

As for these instances in which the equivalent model A performs worse than the equivalent model B, the credibility of the equivalent model A is still very high, due to the fact that active power pulse errors are all lower than 1%.

## Conclusions

In this paper, we proposed an innovative clustering concept for the equivalent modeling of PMSG-based WPPs, which takes into account the impacts of both the DC link chopper protection and the coordinated control of active and reactive powers. The post-fault DC link voltages of PMSGs were calculated based on the pre-fault wind energy and the terminal voltage dip; without any clustering algorithm, the PMSGs were divided into groups according to the diversity in the DC link voltage changes.

As for different POI voltage dips, wind energy and the transfer coefficients of wind energy, simulation results confirmed that the equivalent model has the same effects on power system dynamics as a detailed WPP. The proposed equivalent model is expected to be applicable for the offline electromechanical transient stability in the power system external to the WPP.

For an unbalanced grid fault occurrence, the LVRT control strategy is usually related to the positive quantities. Therefore the proposed equivalent method can also be applied in the unbalanced grid fault.

## Appendix

See Table 4.

**Table 4:** Model parameters

PMSG	Rater power (MW)	2	Rated stator voltage (kV)	0.69
	Rated frequency (Hz)	50	Rated DC bus voltage (kV)	1.1
	R_s_ (pu)	0.0001	X_d_ (pu)	1.5
	X_q_ (pu)	1.5	C (F)	0.01
Grounding transformer	S (MVA)	2.5	X_T_ (%)	6
Main transformer	S (MVA)	150	X_T_ (%)	13.5
Chopper protection	R_cr_ (Ω)		1.2	
Overhead line				
LGJ-240/35	r_0_ (Ω/km)	0.131	x_0_ (Ω/km)	0.372
LGJ-300/220	r_0_ (Ω/km)	0.107	x_0_ (Ω/km)	0.414

## References

[CR1] Abbes M, Allagui M, Hasnaoui OK (2015) An aggregate model of PMSG-based, grid connected wind farm investigation of LVRT capabilities. In: Proceedings of 6th international renewable energy congress, pp 1–6

[CR2] Ali M, Ilie IS, Milanovic JV, Chicco G (2013). Wind farm model aggregation using probabilistic clustering. IEEE Trans Power Syst.

[CR3] Brochu J, Larose C, Gagnon R (2011). Validation of single-and multiple-machine equivalents for modeling wind power plants. IEEE Trans Energy Convers.

[CR4] Cheng Y, Sahni M, Conto J, Huang S, Schmall J (2011). Voltage-profile-based approach for developing collection system aggregated models for wind generation resources for grid voltage ride-through studies. IET Renew Power Gener.

[CR5] Conroy J, Watson R (2009). Aggregate modelling of wind farms containing full-converter wind turbine generators with permanent magnet synchronous machines: transient stability studies. IET Renew Power Gener.

[CR6] Elizondo MA, Shuai L, Ning Z, Samaan N (2011) Model reduction, validation, and calibration of wind power plants for dynamic studies. In: Proceedings of 2011 IEEE power and energy society general meeting, pp 1–8

[CR7] Erlich I, Shewarega F, Feltes C, Koch F, Fortmann J (2012) Determination of dynamic wind farm equivalent using heuristic optimization. In: Proceedings of 2012 IEEE power and energy society general meeting, pp 1–8

[CR8] Fernández LM, García CA, Saenz JR, Jurado F (2009). Equivalent models of wind farms by using aggregated wind turbines and equivalent winds. Energy Convers Manag.

[CR9] Hussein DN, Mater M, Iravani R (2013). A type-4 wind power plant equivalent model for the analysis of electromagnetic transients in power systems. IEEE Trans Power Syst.

[CR10] Jin YQ, Ju P, Pan XP (2014). Analysis on controller aggregation method for equivalent modeling of DFIG-based wind farm. Autom Electr Power Syst.

[CR11] Muljadi E, Ellis A (2010) Final project report WECC wind generator development. Prepared by National Renewable Energy Laboratory, California Inst Energy Environ. http://www.uc-ciee.org/downloads/WGM_Final_Report.pdf

[CR12] Muljadi E, Butterfield CP, Ellis A, Mechenbier J, Hochheimer J, Young R, Miller N, Delmerico R, Zavadil R, Smith JC (2006) Equivalencing the collector system of a large wind power plant. In: Proceedings of 2006 IEEE power engineering society general meeting, pp 1–9

[CR13] Neumann T, Wijnhoven T, Deconinck G, Erlich I (2015). Enhanced dynamic voltage control of type-4 wind turbines during unbalanced grid faults. IEEE Trans Energy Convers.

[CR14] State Grid Corporation of China (2009) Technology rule for connecting wind farm into power grid. Beijing

[CR15] Zhu QL, Han PP, Ding M, Zhang XA, Shi WH (2014). Probabilistic equivalent model for wind farms based on clustering-discriminant analysis. Proc CSEE.

[CR16] Zou J, Chao P, Xu H, Yan Y (2015). A fuzzy clustering algorithm-based dynamic equivalent modeling method for wind farm with DFIG. IEEE Trans Energy Convers.

[CR17] Zubia I, Ostolaza JX, Susperregui A, Ugartemendia JJ (2012). Multi-machine transient modelling of wind farms: an essential approach to the study of fault conditions in the distribution network. Appl Energy.

